# Skin Lesion Segmentation Using an Ensemble of Different Image Processing Methods

**DOI:** 10.3390/diagnostics13162684

**Published:** 2023-08-15

**Authors:** Maria Tamoor, Asma Naseer, Ayesha Khan, Kashif Zafar

**Affiliations:** 1 Department of Computer Science, Forman Christian College, Lahore 54600, Pakistan; mariatamoor@fccollege.edu.pk (M.T.); ayeshakhan@fccollege.edu.pk (A.K.); 2 Department of Computer Science, National University of Computer and Emerging Sciences, Lahore 54770, Pakistan; asma.naseer@nu.edu.pk

**Keywords:** ensemble, dermoscopy, thresholding, CAD, preprocessing

## Abstract

In recent times, there has been a huge increase in the average number of cases of skin cancer per year, which sometimes become life threatening for humans. Early detection of various skin diseases through automated detection techniques plays a crucial role. However, the presence of numerous artefacts makes this task challenging. Dermoscopic images exhibit various variations, including hair artefacts, markers, and ill-defined boundaries. These artefacts make automatic analysis of skin lesion quite a difficult task. To address these issues, it is essential to have an accurate and efficient automated method which will delineate a skin lesion from the rest of the image. Unfortunately, due to the presence of several types of skin artefacts, there is no such thresholding method that can provide a sufficient segmentation result for every type of skin lesion. To overcome this limitation, an ensemble-based method is proposed that selects the optimal thresholding based on an objective function. A group of state-of-the-art different thresholding methods such as Otsu, Kapur, Harris hawk, and grey level are used. The proposed method obtained superior results (dice score = 0.89 with *p*-value ≤ 0.05) as compared to other state-of-the-art methods (Otsu = 0.79, Kapur = 0.80, Harris hawk = 0.60, grey level = 0.69, active contour model = 0.72). The experiments conducted in this study utilize the ISIC 2016 dataset, which is publicly available and specifically designed for skin-related research. Accurate segmentation will help in the early detection of many skin diseases.

## 1. Introduction

Automated visual inspection in medical imaging aims to diagnose different diseases at their early stages. Early detection is crucial for effective treatment and especially for recovery of otherwise deadly diseases. Unless early detection and diagnosis is carried out, the diseases may spread and result in fatality. Abnormal cell development in human skin relies greatly on early detection for diagnosis and treatment where automated visual inspections with computer-aided diagnostic systems complement a human expert examination, as skin lesions are too initially too tiny to be accurately detected by human vision [1,2,3]. Skin cancers due to abnormal skin cell development can be classified into basal or squamous cell carcinoma and melanoma. Melanoma, although rare, causes the most deaths in skin cancer patients. It can spread to deeper tissues and other organs with severe outcomes. Dermoscopy magnifies and eliminates skin reflection [4] for segmentation of skin lesion, pattern recognition, and classification.

The analysis of skin lesions to determine their benign or malignant nature poses challenges due to their similar appearances. There are various limitations, such as ill-defined boundaries, added markers, artefacts from hair, and background variations. When dealing with images that have clear borders and no artefacts, basic thresholding methods with simple objective functions can be applied for segmentation. However, in cases where images exhibit various variations, a well-defined objective function is necessary to determine an optimized thresholding value. Interestingly, previous research has primarily focused on using different machine learning methods, neglecting the utilization of thresholding methods.

In previous studies [5], widely accepted deep learning methods were employed for skin lesion analysis. These methods involved creating ensembles of multiple convolutional neural networks, incorporating multi-scale information, and adopting a multi-task learning framework. The objective behind these approaches was to utilize as much information as possible to achieve robust predictions. Current approaches to pattern recognition make use of DCNNs and ML methods. One major success factor for a DCNN model is the availability of data for fine tuning of the model and accurate predictions. As opposed to other domains where the datasets consist of tens of millions [6], skin lesion datasets have only thousands of entries. In contrast to other datasets, skin lesions images have a higher coupling among different classes and a lower cohesiveness within images of the same class [7]. A melanoma may have more similarities with a carcinoma rather than other melanoma images. Such similarities within class and across different class variations make skin lesion classification a challenge. Another issue is the overlap between skin lesion and healthy skin or harmless artefacts such as moles or vitiligo which are irrelevant but interfere with the classification. A recent study has proposed an ensemble-based neural network using individual predictors from the constituent models for better convergence and optimal results improving the general performance [5].

**Figure 1:**
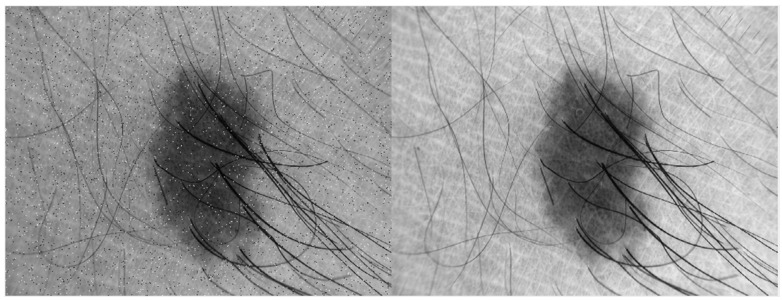
Applying median filter for skin image to remove noise.

We propose an ensemble-based thresholding method for better segmentation. As skin lesions images have ill-defined boundaries and added hair with numerous other artefacts Figure 1, an intelligent objective function is defined by ensemble learning for optimised thresholding. We have used five thresholding methods, namely, Otsu, active contour, Kapur, grey threshold, and Harris hawk.

The organization of the paper is as follows, Section 2 summarises the existing techniques for skin lesion segmentation, and Section 3 provides an insight into the proposed methodology. Results are portrayed in Section 4. A comprehensive discussion is provided in the subsequent Section 5. Finally, Section 6 concludes the research regarding skin lesion segmentation using the novel approach.

## 2. Literature Review

Skin lesion segmentation is an important technique for the early detection and diagnosis of different types of skin diseases, including melanoma and other types of skin cancer. Due to variations in lesion appearance, texture, and surrounding skin, the correct and efficient segmentation of skin lesions is a challenging task. To address this challenge, authors have explored various image processing methods to enhance segmentation performance.

The authors of [8] presented an automatic system for classifying skin lesions as benign, melanoma, and malignant. The system implements the ABCD rule, which analyses asymmetry, border irregularity, colour variation, and diameter as key features. Preprocessing, feature extraction, and classification using k-nearest neighbours (KNN) and support vector machine (SVM) were employed. Another research [9] introduces an approach for segmenting skin lesions in demographic images that is novel. The proposed method combined adaptive thresholding and colour model normalization techniques to solve the issues during segmentation, by variations in illumination and colour in dermoscopic images. The results clearly showed the accurateness and robustness of the proposed approach for segmentation of skin lesions. The authors concluded by stating that the combination of adaptive thresholding and colour model normalization was effective in accurately segmenting skin lesions.

There is another study [10] that explored various metrics for evaluation image segmentation techniques specifically applied to skin lesions. The study focused on six metrics: Mathews correlation coefficient, Dice coefficient, accuracy, specificity, sensitivity, and the Jaccard index. These metrics are commonly used to assess the performance of segmentation algorithms in the field of medical image analysis. The paper discussed the mathematical formulation and interpretation of each metric, providing insight into their strengths and limitations. The authors emphasized the importance of using multiple metrics to comprehensively evaluate segmentation results. The paper concluded that a combination of these metrics provides a more comprehensive assessment of the segmentation accuracy and can aid in the development and improvement of skin lesion algorithms.

Classification algorithms have been widely used for classifying lesions in many research articles. One such article [11] focused on the application of decision trees and random forest algorithms for classifying skin lesions. The authors explored these techniques to enhance the accuracy of classification for skin lesion. They compared the performance of decision trees and random forests using various evaluation metrics. Both algorithms are known to effectively classify skin lesions. The random forest algorithm outperformed the decision tree in terms of accuracy and robustness.

Convolution neural networks (CNN) have emerged as a powerful tool for skin lesion segmentation during previous years. In 2018, [12] focused on multimodal skin lesion classification using deep learning. This study explored the use of various imagine modalities, including dermoscopy, clinical photography, and reflectance confocal microscopy, for improved classification accuracy. Deep learning models are trained on a large dataset of skin lesion images, and the results demonstrate the effectiveness of the multimodal approach in enhancing classification performance. The study [13] proposes a skin lesion method using object-scale-oriented fully CNN (FCNNs). The approach focused on capturing object-scale information to improve segmentation accuracy and highlights the importance of considering object scale during the segmentation of skin lesion using deep learning techniques. A very comprehensive survey was provided by [2] for skin image techniques using convolution neural networks (CNNs). Multiple CNN architectures, such as U-Net, FCN, and DeepLab, were compared and analysed in terms of accuracy, robustness, and computational efficiency. Experimental results provided insights into the strengths and weaknesses of each technique and highlight their performance variations across different skin image datasets.

Local binary pattern (LBP) clustering has also been used by [14] for skin lesion segmentation. This study summarised the analysis of different segmentation methods based on LBP. The effectiveness of LBP clustering was evaluated using various metrics, including the Dice coefficient, sensitivity, and specificity.

Ensemble methods are a type of technique that combines multiple individual models to improve predictive performance and robustness. The challenging problem of skin lesion segmentation has also been solved by exploring ensemble approaches. Different ensemble-based approaches have been explored in the literature [15]. Deep learning and machine learning algorithms were used in an ensemble to classify skin lesions in this study. They have combined the strengths of both techniques to improve accuracy and reliability, and [16] proposed an ensemble approach of fully convolution neural networks (FCNNs) for computerized segmentation of skin lesions. The ensemble combines multiple FCNN models to demonstrate the effectiveness of ensemble approach. In a similar manner [17], used an ensemble approach that combines multiple deep learning models for improved accuracy in disease detection and classification. For classification, an RF classifier-based DCNN was implemented because it uses ensemble models to classify pattern extracted features.

The challenges in skin lesion segmentation have generated certain gaps that the existing literature has still not addressed [18,19,20]. A few key areas where the existing approaches can be improved include the lack of diversity of datasets, limited standard evaluation metrics, class imbalance problem, robustness against diversity in imaging conditions, incorporating clinical contexts, and real-time segmentation. By consolidating these gaps, the technique can be advanced and made more robust, accurate, and clinically useful segmentation approaches can be developed.

The major research contributions are mentioned below:

- For early detection of various skin diseases, a novel skin lesion segmentation method is proposed to automate skin lesion segmentation. The proposed method maintains novelty by employing an ensemble-based method to determine the optimal thresholding, This selection is based on an objective function, aiming to automate the process of skin lesion segmentation.
- The ensemble-based novel approach accurately and efficiently automates the segmentation process of skin lesion from the rest of the image. Furthermore, it successfully overcomes the limitations caused by various artefacts.
- The proposed method provides optimal thresholding for all types of skin images, which is not possible using only one method for all types. Hence, it does not require any training of the data which makes it more efficient then deep learning and other classification models.
- The proposed methodology is tested on a benchmark skin dataset ISIC 2016, and provides superior results (Dice score = 0.89 with *p*-value ≤ 0.05) as compared to state-of-the-art methods.

## 3. Methodology

Many algorithms have been proposed for accurate skin lesion segmentation, which are later used for different skin diseases. In this paper, a group of thresholding methods are used for improved segmentation results. The complete methodology is divided into following steps:

1. Data selection;
2. Preprocessing;
3. Binary Conversion using optimal thresholding value;
4. Postprocessing for skin lesion segmentation.

### 3.1. Data Selection

**Figure 2:**
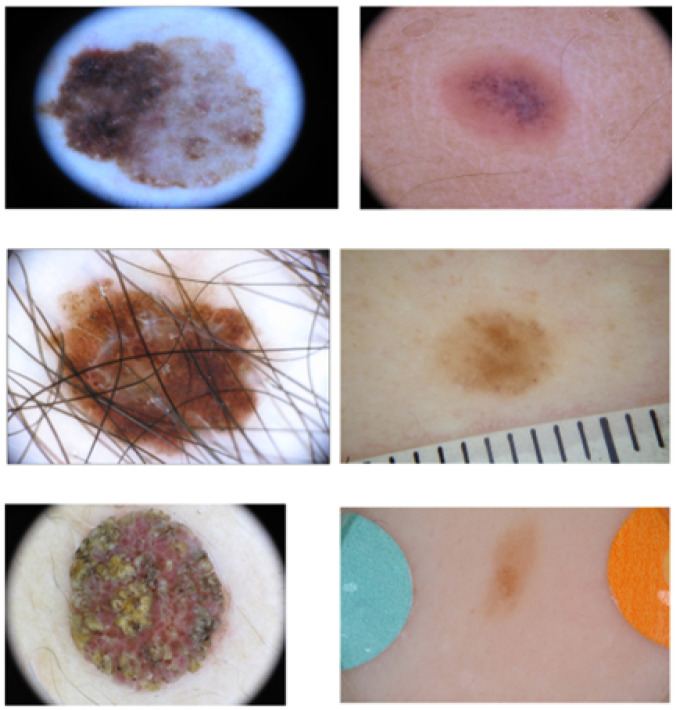
Skin images with different artefacts from ISIC 2016 dataset [21].

The initial stage involves extracting images from the ISIC 2016 dataset [21], a comprehensive collection of dermoscopic images. Acquiring dermoscopic images is crucial for our research as they facilitate image segmentation and the identification of potentially malignant lesions. This dataset offers a wide range of skin lesion images, including numerous challenging instances characterized by issues such as ill-defined boundaries, low contrast, presence of hair artefacts, markers, and intensity inhomogeneity. The skin dataset comprises a total of 900 skin lesion images exhibiting various types of artefacts and the ground truth is available for each skin lesion. Figure 2 shows a variety of skin images from the dataset, highlighting the presence of distinct challenging artefacts.

### 3.2. Preprocessing

Preprocessing techniques in skin lesion segmentation are crucial for improving image quality, reducing noise, enhancing contrast, removing artefacts, and standardizing images [22,23]. These preprocessing steps contribute to more accurate and reliable segmentation results, which help in improved diagnosis of different diseases [22].

**Figure 3:**
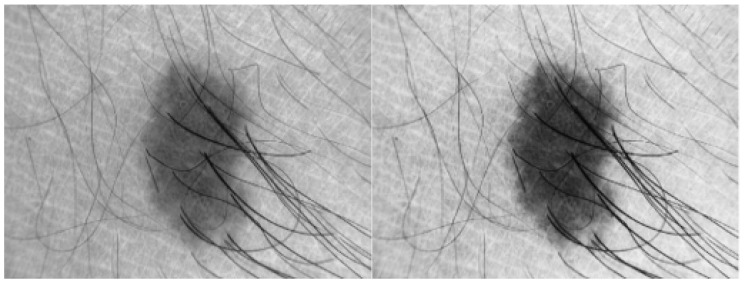
Applying image enhancement for skin image to improve contrast.

In this research, basic image processing steps are performed to enhance image quality and remove different artefacts. First, all images are resized to 56 × 56 dimensions to make program efficient. To remove noise from the input skin image, median filter is used which is shown in Figure 1, however this filter preserves edges of the image. The contrast of the input image is improved using *imadjust* (using a MATLAB function) as shown in Figure 3.

After enhancing image quality of input image, the next step is the most crucial step, that is, finding the optimal threshold value for the binarised image.

### 3.3. Binary Conversion Using Optimal Thresholding Value

In research, different thresholding methods have been used to convert image into binary form; however, there is no single method which is sufficient for finding the optimal thresholding value for every type of image. Certain methods excel in images with distinct edges, whereas others are more suitable for handling low-contrast images.

In this paper, an ensemble approach is adopted, incorporating a group of four thresholding methods and one snake model. An objective function is utilized to assess which method yields the most optimal value for binarising each input image. This approach works similar to a winner-takes-all approach which picks the best method according to the input image. The ensemble model uses the following thresholding methods for analysis:

1. Hawk thresholding;
2. Kapur thresholding;
3. Otsu thresholding;
4. Gray thresholding;
5. Active contour model.

**Figure 4:**
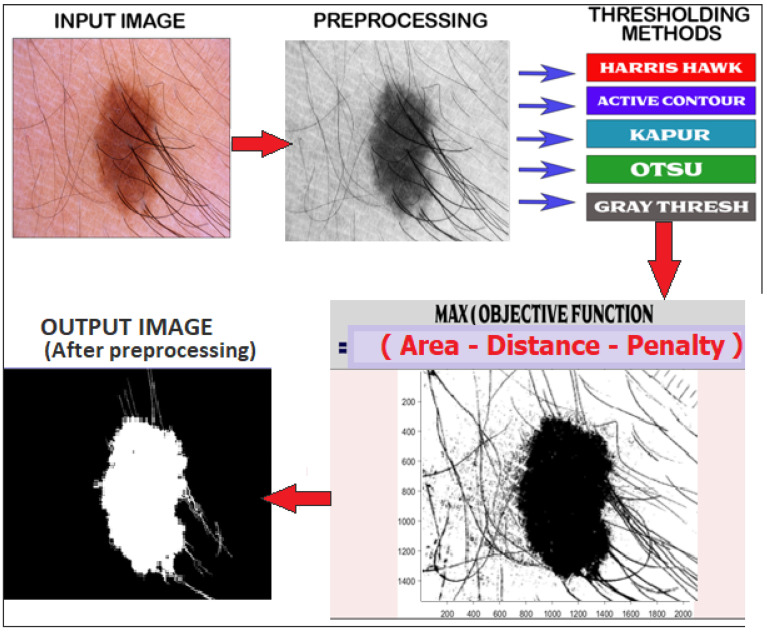
Overall diagram of proposed method for skin lesion segmentation.

Figure 4 illustrates the comprehensive workflow of the proposed method, which integrates various thresholding methods and the active contour model (ACM) to determine the optimal value for binary conversion. This optimal binary conversion value is then utilized for accurate segmentation of the input image. Maximum value of an objective function is used to determine optimal thresholding value among all methods. The objection function is given in the equation.

Brief description of each model is given below:

#### 3.3.1. Harris Hawk Thresholding Method

Harris hawk optimization [24] is used to binarise input image using a fitness function which is based on cross entropy. Cross entropy is calculated using entropy of the input image and the automated segmented image. This method computes information distance I = $I_{1},I_{2},\ldots I_{n}$ between both images which is defined in Equation (1)

(1) $$Dist\left(I,S\right)=\sum_{j=1}^{N}=I_{j}log\frac{I_{j}}{S_{j}}$$

#### 3.3.2. Kapur Thresholding Method

Kapur’s entropy, proposed by Akay [25], is recognized as one of the most effective threshold selection methods for real-world images due to its ability to preserve uniformity and shape measures. This method determines threshold values in a manner that ensures the preservation of grey-level moments from the input image to the output image. Kapur’s entropy consistently yields positive probabilities and results in a global maximum for entropy. In Kapur’s entropy $th_{1},th_{2}\ldots L-1$ represents different levels of grey image and $g_{1},g_{2},\ldots L-1$ represents grey levels of non-local mean image. Its entropy can be defined as

(2) $$\phi \left(g,th\right)=\sum_{i=0}^{g_{1}}\sum_{j=0}^{th_{1}}\frac{P_{ij}}{P_{1}}ln\frac{P_{ij}}{P_{1}}-\sum_{i=th_{1}+1}^{g_{2}}\sum_{j=th_{1}+1}^{th_{2}}\frac{P_{ij}}{P_{2}}ln\frac{P_{ij}}{P_{2}}-\ldots -\sum_{i=g_{L-2}+1}^{g_{L-1}}\sum_{j=th_{L-2}+1}^{th_{L-1}}\frac{P_{ij}}{P_{L-1}}ln\frac{P_{ij}}{P_{L-1}}$$

where $P_{ij}$ is defined as

$P_{ij}=\frac{hist(i.j)}{M\times N}$, hist is the count of total pixels at a given point using histogram and M and N are dimensions.

#### 3.3.3. Otsu Thresholding Method

This method involves analysing the histogram of the input image and segment region of interest (ROI) by minimizing the variance within each class. The fundamental concept of this method is to divide the image histogram into two distinct clusters, with a threshold determined by minimizing the weighted variance of these classes, represented as $\sigma_{w}^{2}\left(th\right)$.

The computation equation for the weighted variance, denoted as $\sigma_{w}^{2}\left(th\right)$, can be expressed as follows:

$\sigma_{w}^{2}\left(th\right)=w_{1}\left(th\right)\sigma_{1}^{2}\left(th\right)+w_{2}\left(th\right)\sigma_{2}^{2}\left(th\right)$. In this equation, $w_{1}\left(th\right)$ and $w_{2}\left(th\right)$ represent the probabilities of the two classes divided by a threshold th, where the threshold value th ranges from 0 to 255.

#### 3.3.4. Gray Thresholding Method

Gray thresholding is a highly efficient and commonly employed technique for image segmentation. This method incorporates spatial image information in threshold selection which is based on the distribution of measurement of a specific feature of the objects which is present at every threshold level of the histogram. The basic principle behind the object feature histogram can be described as follows:

For each grey-level threshold level th, generate the binary image $I_{th}$ and calculate a feature measurement $f(I_{th})$, such as the average input size. Based on this feature measurement, a histogram h[th] for the feature is defined as h[th] ← $f(I_{th})$.

#### 3.3.5. Active Contour Model

Active contour models (ACMs) are extensively used for medical image segmentation due to their robustness against ill-define boundaries, low-contrast, and intensity inhomogeneous artefacts [26,27,28,29]. ACMs operate by minimizing an energy function, allowing the evolution of a segmenting curve that accurately delineates a region of interest (ROI) from other objects in the image. Many variations of ACMs have been proposed but the main energy functional to divide image into foreground and background, is based on the Chan Vese model (CV) which can be described using two fitting energies as shown in Equation (3):

(3) $$\int_{In(Contour)}u_{0}-c_{1}^{2}dx+\int_{Out(Contour)}u_{0}-c_{2}^{2}dx$$

All these aforementioned methods are implemented to get optimal thresholding value for one specific input image.

#### 3.3.6. Objective Function

Five different thresholding values are obtained from above mentioned methods. An objective function is implemented to obtain best optimal value out of these five thresholding values. This objective function is based on the prior knowledge of the skin lesion images and it is specific for skin datasets. This objective function comprises of area of the ROI, distance of ROI from the centre of the image and some penalty terms. Area and Euclidean distance are normalized. The overall objective function can be formalized as follows given in Equation (4):

(4) $$Objective\_function{\left(th\right)}_{max}=Area\left(C\right)-Distance\left(C\right)-penalty\left(C\right)$$

For each aforementioned method, largest component C will be selected for consideration among all the binarized objects. Area(C) is the number of pixels covered by C, Distance (C) is calculated using the Euclidean distance from the centre of C to the centre of the input Image I. Penalty(C) is employed to evaluate whether the outcome of thresholding an image corresponds to an entirely black or entirely white region. S(x,y) represents the pixel’s value located at the row x and column y of the thresholded binary image and A × B is the size of the input dermoscopic image. The penalty is calculated as given in Equation (5):

(5) $$\;Penalty\left(C\right)=\left\{\begin{array}{c} \mathrm{if}\;\sum \;S(x,y)\;==\;A\;\times \;B\;\;\;\;\mathrm{penalty}\;=\;\infty \\ \mathrm{if}\;\sum \;S(x,y)\;==\;0\;\;\;\;\;\;\;\;\;\;\;\;\mathrm{penalty}\;=\;\infty \\ \mathrm{otherwise}\;\;\;\;\;\;\;\;\;\;\;\;\;\;\;\;\;\;\;\;\;\mathrm{penalty}\;=\;0 \end{array}\right.$$

#### 3.3.7. Postprocessing for Skin Lesion Segmentation

**Figure 5:**
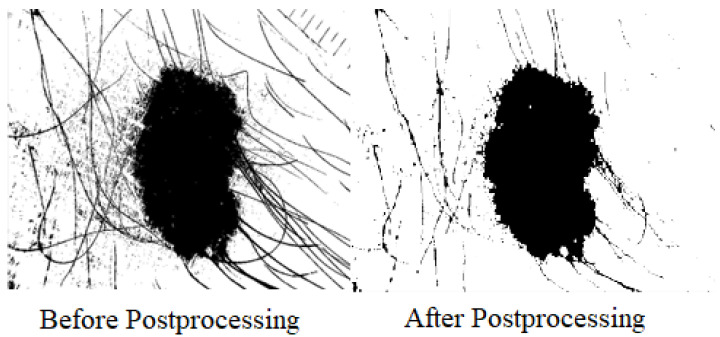
Effect of Postprocessing on binary image.

After obtaining one output binary image after applying Equation (4), some postprocessing is performed for shrinkage and removal of small openings from the binary image. The postprocessing has removed some of the artefacts with some morphological processes, i.e., erosion for shirking and dilation followed by erosion for hole filling, for the ultimate test by feeding segmented images to pattern recognition models for classification.The effect of postprocessing is described in Figure 5.

### 3.4. Evaluation of Proposed Model

For evaluation, different metrics are used to compare results comprehensively. Dice score (DSC), Jaccard index (JI), specificity, and sensitivity are used for all comparisons which are defined below:

(6) $$Dice\;\;Score\;\left(DSC\right)=2\times \frac{A\cap G}{A+G}$$

where A is the binary image produced by proposed method and G is the ground truth.

(7) $$Jaccard\;\;Index\;\left(JI\right)=\frac{DSC}{2-DSC}$$

(8) $$Specificty\left(SP\right)=\frac{TN}{TN+FP}$$

where FP and TN are false positive and true negative, respectively. It calculates rate of correct Negative examples.

(9) $$Sensitivity\;\left(SE\right)=\frac{TP}{TP+FN}$$

where FN and TP are false negative and true positive, respectively. It calculates rate of correct positive examples. To evaluate the significance of the results generated by the proposed model and five other algorithms (Otsu, Kapur, grey-level, Harris hawk, and ACM), a two-sample *t*-test is utilized. The test is conducted with a confidence interval of 95% (p≤0.05).

## 4. Experiments

The proposed ensemble based model is evaluated extensively on large dataset of ISIC 2016 which contains diverse type of skin images. Results of ensemble based model are compared with all state-of-the-art threhsolding models and ACM to analyse robustness and accuracy of this approach. All the experiments are performed on MATLAB 2017a.

### Results and Comparisons

**Figure 6:**
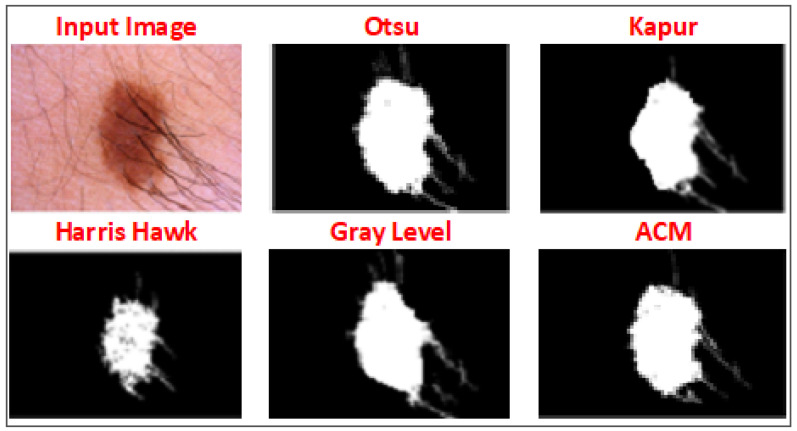
Output of different thresholding methods and ACM and selection of best method for the input image.

Equation (4) represents the objective function, which effectively determines the optimal thresholding value to binarise the input image, resulting in improved segmentation outcomes. In Figure 6, the outputs of various methods for a specific image are shown. Among these methods, Otsu generated the most favourable binary image for the region of interest (ROI). Notably, the proposed objective function also identified and selected the same thresholding value for binarisation, aligning with the superior performance of Otsu’s method.

**Figure 7:**
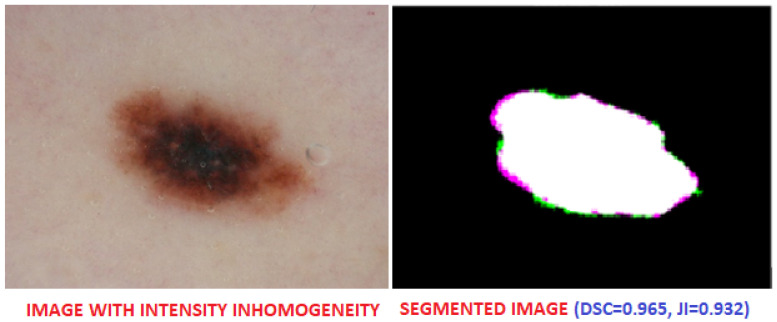
Output of skin image with intensity inhomogeneity.

Figure 6 shows segmentation of a challenging skin image due to presence of hair in the image. Similarly, Figure 7 shows a skin image with severe intensity inhomogeneity, and the proposed method produced an accurate segmentation of ROI with Dice score = 0.965.

The proposed method is validated on all skin images and the output of above mentioned methods are analysed in detail. Table 1 presents the outcomes obtained for various images, demonstrating the effectiveness of the ensemble-based model. This model, which relies on an objective function, adeptly selects the optimal thresholding value for skin lesion segmentation. The results clearly illustrate the model’s ability to accurately determine the threshold, leading to successful segmentation of skin lesions across different images.

All thresholding models (OTSU, Harris hawk, grey level, and Kapur) and the active contour model are good for different types of segmentation but for diverse type of input images with different challenges, they failed to produce overall good Dice scores and other values. In contrast, the proposed ensemble based model produced promising results. The comparison of different thresholding methods and state-of-the-art methods (DOLHGS [30], EDB-CNN [31], Auto-ED [32], LIN [33], FCN-8 s [34], and U-Net [35]) for skin lesion segmentation using same datasets are given in Table 2.

## 5. Discussion

In skin lesion segmentation, finding an optimal thresholding method can be challenging due to the presence of inhomogeneity, noise, and other artefacts in the images. Different research studies have employed various thresholding methods, but no single method is universally effective for all types of images. Some methods work well for images with distinct edges, whereas others are more suitable for handling low-contrast images. To address this issue, an ensemble approach has been adopted in this research, which incorporates a group of thresholding methods. The ensemble model aims to determine the best thresholding value for binarising each input image by evaluating the performance of multiple methods. The four thresholding methods used in this ensemble are Hawk thresholding, Kapur thresholding, Otsu thresholding, and grey thresholding. These methods have been chosen based on their effectiveness in segmenting skin lesions. Additionally, an active contour model, also known as a snake model, is included in the ensemble to further improve the segmentation accuracy.

To evaluate and compare the performance of these thresholding methods, an objective function is implemented. This objective function is specifically designed for skin lesion datasets and incorporates prior knowledge about these images. It takes into account several factors, including the area of the region of interest (ROI) obtained from each thresholding method, the distance of the ROI from the centre of the image, and certain penalty terms. By combining these factors, the objective function assesses the quality of the segmented regions produced by each method and identifies the optimal thresholding value.

By utilizing an ensemble approach and an objective function tailored for skin lesion images, this research aims to overcome the limitations of individual thresholding methods and enhance the accuracy of segmentation. The ensemble model leverages the strengths of each method and combines their results to achieve a more robust and reliable segmentation outcome. This approach recognizes the diverse characteristics and challenges present in skin lesion images and provides a more comprehensive solution for accurate segmentation.

This research can be easily extendable for other medical imaging modalities such as cardiac magnetic resonance imaging (CMR), ultrasounds, and brain scans. By utilizing the same model, the only necessary adjustment would involve adapting the objective function to suit the specific requirements of any new imaging modality.

Table 2 presents the results of various algorithms for skin lesion segmentation based on different evaluation metrics, and compares these state-of-the-art approaches with the proposed model. The Dice score and Jaccard index provide insights into the overlap between the predicted segmentation and the ground truth. Higher values indicate better segmentation accuracy, and the proposed model achieves a Dice score of 0.89 and a Jaccard index of 0.85, which are the highest among all methods and indicates a strong agreement between the predicted and ground truth segmentation. As sensitivity represents the true positive rate, measuring how well the algorithm detects positive class (skin lesion) pixels, the proposed model achieves the highest sensitivity of 0.92, indicating a high capability to identify and capture skin lesions. Finally, specificity measures the true negative rate, indicating how well the algorithm excludes background pixels from the segmentation and the proposed model achieves a specificity of 0.93 as well thus, indicating a high ability to accurately identify background pixels. Overall, the proposed model consistently shows competitive performance across all evaluated metrics, achieving the highest Dice score, Jaccard index, sensitivity, and specificity among the listed algorithms.

## 6. Conclusions

A new model is proposed for an accurate skin lesion segmentation from dermoscopic images. A diverse type of dataset (ISIC 2016) is used which contains skin images with different types of artefacts. Several segmentation methods are used to perform this task but different images require different thresholding values for best segmentation results. In this paper, an ensemble of five different models are used to obtain an optimal thresholding value based on an objective function. This ensemble approach ensures a robust selection process for determining the ideal thresholding value. Comprehensive experiments are performed on the entire dataset, and it is observed that using a group of different methods produced superior results in comparison to any single state-of-the-art segmentation method. By employing this ensemble-based approach and conducting comprehensive experiments, we demonstrate the efficacy of our model in accurately segmenting skin lesions. This contributes to improved diagnostic and treatment processes, providing valuable insights for medical professionals in the field of dermatology.

## Figures and Tables

**Table 1:** Dice scores of different models for different types of skins images. Last column represents method selected on the basis of proposed model.

Images	Otsu	ACM	Kapur	Harris Hawk	Gray Level	Method
ISIC_0000088	0.90832	0.89734	**0.9914**	0.71343	0.90815	**Kapur**
ISIC_0000092	**0.98213**	0.95409	0.97993	0.89431	0.98115	**Otsu**
ISIC_0000098	0.85285	**0.93385**	0.84577	0.92963	0.85246	**ACM**
ISIC_0000099	0.81256	0.81607	**0.84607**	0.43026	0.81264	**Kapur**
ISIC_0000107	0.8167	0.80181	**0.90509**	0.59085	0.81662	**Kapur**
ISIC_0000111	0.88377	0.87495	0.854	**0.75201**	0.88377	Harris Hawk
ISIC_0000115	0.94672	0.90109	0.93695	0.69199	**0.94614**	**Gray Level**
ISIC_0000117	**0.75444**	0.73468	0.74833	0.49084	0.70436	**Otsu**
ISIC_0000138	0.91302	0.91033	0.89869	0.77758	**0.92325**	**Gray Level**
ISIC_0000141	0.85414	0.84517	**0.96452**	0.65194	0.85254	**Kapur**
ISIC_0010037	0.70562	0.67803	**0.9055**	0.46567	0.7061	**Kapur**
ISIC_0010077	**0.72987**	0.705	0	0	0.72977	**Otsu**
ISIC_0010088	**0.96529**	0.8419	0.86761	0.77007	0.96451	**Otsu**
ISIC_0010089	0.84911	0.80495	0.82336	0.67474	**0.84937**	**Gray Level**
ISIC_0010229	0.80148	0.84719	0.90858	**0.94514**	0.80126	**Harris Hawk**
ISIC_0000052	0.8724	0.87699	0.83467	**0.76026**	0.87218	Harris Hawk
ISIC_0011325	0.78548	0.82394	**0.86398**	0.36901	0.78414	**Kapur**
ISIC_0011333	0.87469	0.88044	**0.93349**	0.59295	0.87488	**Kapur**
ISIC_0011336	0.73491	**0.78523**	0.75955	0.61968	0.73502	**ACM**
ISIC_0002107	0.92998	0.92994	**0.9842**	0.877445	0.93	**Kapur**
ISIC_0009882	0.79709	0.80319	**0.90163**	0.21944	0.79736	**Kapur**
ISIC_0009885	**0.86217**	0.77839	0.80061	0.70652	0.8621	**Otsu**
ISIC_0009891	0.84164	**0.85852**	0.85295	0.74451	0.84195	**ACM**
ISIC_0000003	**0.95172**	0.94487	0.96831	0.5486	0.94172	**Otsu**
ISIC_0000012	**0.88946**	0.96935	0.96605	0.89799	0.88959	Otsu
ISIC_0000013	0.94618	0.94372	**0.95312**	0.8475	0.94632	**Kapur**
ISIC_0000014	0.8772	**0.88966**	0	0.45025	0.87773	**ACM**
ISIC_0000015	0.81411	0.78472	**0.81964**	0.57459	0.81383	**Kapur**
ISIC_0000020	0.80277	0.75279	**0.81669**	0	0.8026	**Kapur**
ISIC_0011129	0.80976	**0.86078**	0.78515	0.2975	0.80902	**ACM**
ISIC_0000022	0.62025	**0.63056**	0.58314	0.21623	0.62185	**ACM**
ISIC_0000023	0.87467	0.8745	**0.97339**	0	0.87462	**Kapur**
ISIC_0000027	0.74721	0.75558	**0.85704**	0	0.74662	**Kapur**
ISIC_0000197	0.85613	0.7689	**0.84077**	0.4621	0.83616	**Otsu**
ISIC_0010553	0.84222	0.83792	**0.92108**	0.76132	0.84271	**Kapur**
ISIC_0011150	0.71494	**0.72044**	0.70996	0.63126	0.71944	**ACM**
ISIC_0011110	0.80332	0.80631	**0.84124**	0.7108	0.80349	**Kapur**
ISIC_0011112	0.79152	0.77896	0.76354	**0.87658**	0.79332	**Harris Hawk**

**Table 2:** Comparison of different methods for skin lesion Segmentation using the ISIC 2016 dataset.

Algorithms	Dice Score	Jaccard Index	Sensitivity	Specificity
**Otsu**	**0.69**	**0.58**	**0.83**	**0.94**
**Active Contour**	**0.72**	**0.61**	**0.76**	**0.92**
**Kapur**	**0.72**	**0.62**	**0.77**	**0.94**
**Harris Hawk**	**0.50**	**0.39**	**0.69**	**0.84**
**Gray Thresh**	**0.69**	**0.58**	**0.80**	**0.91**
**DOLHGS**	**0.87**	**0.85**	**0.87**	**0.88**
**EDB-CNN**	**0.87**	**0.85**	**-**	**-**
**Auto-ED**	**0.82**	**0.79**	**0.71**	**0.81**
**LIN**	**0.83**	**0.80**	**0.77**	**0.89**
**FCN-8 s**	**0.78**	**0.76**	**0.70**	**0.82**
**U-net**	**0.86**	**0.84**	**0.76**	**0.92**
**Proposed Model**	**0.89**	**0.85**	**0.92**	**0.93**

## Data Availability

All data are publicly available and taken from ISIC skin database.
